# Pyrolysis of Kraft Lignin: The Effect of HZSM‑5 and HY-340 Catalysts and Torrefaction Pretreatment

**DOI:** 10.1021/acsomega.5c03569

**Published:** 2025-06-30

**Authors:** Anderson L. de Menezes, Alvaro E. C. Souza, Daniel A. Cerqueira, Cássia R. Cardoso, Luiz G. M. Vieira

**Affiliations:** † Faculty of Chemical Engineering, 28119 Federal University of Uberlândia, Uberlândia, Minas Gerais 38408-100, Brazil; ‡ Multicenter Chemistry Graduate Program of Minas Gerais State, 74348 Federal University of Triângulo Mineiro, Uberaba, Minas Gerais 38064-200, Brazil; § Department of Chemistry, 74348 Federal University of Triângulo Mineiro, Uberaba, Minas Gerais 38064-200, Brazil; ∥ Department of Food Engineering, 74348 Federal University of Triângulo Mineiro, Uberaba, Minas Gerais 38064-200, Brazil

## Abstract

The study aimed to evaluate the effect of HZSM-5 zeolite, HY-340 niobic acid, and torrefaction temperature on the deoxygenation of pyrolysis vapors from the catalytic pyrolysis of kraft lignin (493, 533, and 573 K) to produce aromatic hydrocarbons. The analytical pyrolysis (723, 823, and 923 K) of raw Kraft lignin at different catalyst/biomass ratios (1:1, 5:1, and 10:1) and without catalysts was performed. The pyrolysis vapor from analytical pyrolysis presented high levels of oxygenated compounds, mainly phenolics. The maximum production of phenolic compounds was 74% at 923 K for raw Kraft lignin. Catalytic analytical pyrolysis provided deoxygenation of pyrolysis vapors. HZSM-5 zeolite reached a maximum production of aromatic hydrocarbons of 57.84% with a catalyst/biomass ratio of 10:1 at 923 K. For HY-340 niobic acid, the production of aromatic hydrocarbons was 87.24 and 86.75% at 823 and 923 K, respectively, with a catalyst/biomass ratio of 10:1. The factorial experimental design showed that the maximum catalyst/biomass ratio provided the highest percentage of aromatic hydrocarbons (%HCA). For the HZSM-5 zeolite, the maximum %HCA was 41.95, 53.72, and 92.84% for torrefied lignin at 493, 533, and 573 K, respectively. For HY-340 niobic acid, the maximum %HCA values were 29.29, 50.02, and 90.02% at 493, 533, and 573 K. Fast pyrolysis in a bubbling fluidized bed reactor led to a higher production of phenolic compounds (78.15%) may be due to the longer residence time in the reactor. HZSM-5 zeolite and HY-340 niobic acid catalysts can promote deoxygenation reactions and increase the selectivity for aromatic hydrocarbons.

## Graphical abstract

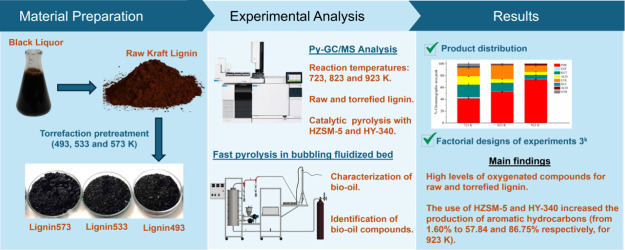

## 1 Introduction

The increasing demand for renewable energy, driven by population growth, industrial activity, and climate issues, can be partially met by forest biomass. This alternative has great potential as a clean and renewable energy source to replace fossil fuels. Biomass energy is neutral in the carbon cycle since the carbon dioxide released into the atmosphere during its combustion is captured in the next biomass cycle through photosynthesis.^1–3^

Lignocellulosic biomass is composed of cellulose, hemicelluloses, and lignin, with extractives and inorganic salts present to a lesser extent. Lignin is the world’s second most abundant naturally occurring complex organic material. It is usually considered a residue with a low value added. However, it can generate high-value-added products, such as synthesis gases, carbon fibers, phenolic compounds, and hydrocarbons. The pulp and paper industries are the traditional source of Kraft lignin, a byproduct from the well-established Kraft pulping process, the most widely used for pulp production.^4,5^

The Kraft pulping process produces black liquor composed of solubilized hemicellulose, lignin, NaOH, and Na_2_S. Kraft lignin can be isolated by acid precipitation and ultrafiltration membrane technology or organic solvents. It has a high calorific value (between 22.5 and 28.5 MJ/kg) and is widely used to meet internal energy demands.^6,7^ Each year, the global production of Kraft lignin ranges from 50 to 70 million tons in pulp and paper mills, with production expected to increase to 225 million tons per year by 2030.^4,8^ However, less than 2% of Kraft lignin is converted into fuels or high-value-added materials, such as dispersants, adhesives, surfactants, or antioxidants in plastics and rubbers. Thus, exploring the potential of this renewable resource is challenging since it can produce valuable functional molecules for the chemical industry.^9,10^

Lignin can be valued through thermochemical and biological processes, among others. Pyrolysis is the thermal degradation of biomass under total or partial absence of oxygen and at temperatures between 673 and 973 K to produce solid (biochar), liquid (bio-oil), and gaseous (noncondensable gases) products. Biomass presenting a high moisture content, low calorific value, and hygroscopicity may hinder its use in pyrolysis. Torrefaction constitutes a thermal pretreatment process to improve the characteristics of biomass (such as concentration of the carbon content, reduction of the oxygen content, and higher grindability) for direct use in pyrolysis. This process is often operated at a low heating rate, under atmospheric pressure, at a temperature ranging between 473–573 K, and in an inert atmosphere. It converts the most unstable oxygenated functions, decreasing the O/C ratio and increasing the calorific value.^11–13^

Bio-oil is the main product of fast pyrolysis. It can be used as fuel or a source of high-value-added chemical products. Generally, fast pyrolysis occurs at temperatures of around 773 K, with high heating rates, short vapor residence time, and rapid vapor cooling. Bio-oil from fast pyrolysis can be used as fuel directly or after treatment to generate energy, heat, biofuels, and chemicals.^14^ However, it is usually composed of several oxygenated compounds, limiting its direct use as a fuel due to the undesirable properties related to these compounds. Catalytic pyrolysis becomes a viable process to deoxygenate bio-oil by introducing catalysts into the pyrolysis process. It is an adaptation of fast pyrolysis to improve the bio-oil quality. In the literature, several catalysts have shown effectiveness in deoxygenation during the pyrolysis process. Among these catalysts, HZSM-5 zeolite and HY-340 niobic acid stand out.^15,16^ During catalytic fast pyrolysis, biomass is rapidly pyrolyzed, generating vapors as primary products with predominantly oxygenated compounds. These compounds enter the catalyst pores and are catalytically converted through a series of reactions, such as deoxygenation, dehydration, decarbonylation, and decarboxylation, among others, to generate the products in which oxygen is primarily removed as CO, CO_2_, and H_2_O.^17,18^

Analytical pyrolysis (micropyrolysis) can be used to analyze the products from fast pyrolysis. Py-GC/MS (pyrolysis-gas chromatography/mass spectrometry) is an analytical method to evaluate the composition of the pyrolysis vapors from the thermal degradation of biomass to form bio-oil. This process effectively detects pyrolytic products by comparing the total chromatographic peak areas obtained during the pyrolysis process under different conditions.^19,20^ Analytical pyrolysis can provide important information about the catalytic effect of the thermal degradation of biomass. Thus, catalytic analytical pyrolysis is a technique to investigate the effect of catalysts on the production of hydrocarbons and other desirable chemical products through the thermal degradation of biomass before conducting experiments in larger-scale equipment.^21^

Several studies have applied Py-GC/MS to evaluate the products from biomass pyrolysis. Mishra and Vinu^22^ studied the fast pyrolysis of residual peanut shells. They observed an increase in the production of hydrocarbons (14.22–24.26%) and alcohols (1.41–9.49%) and a decrease of phenols (19.13–18.67%) by increasing temperature from 723 to 923 K. Mishra et al.^23^ also found that lower temperatures (573 K) increased products rich in oxygen and nitrogen and higher temperatures (823 and 873 K) increased hydrocarbons and alcohols in the pyrolysis of dahlia flowers. Vichaphund et al.^24^ characterized the catalytic pyrolysis of Jatropha waste. The authors reported that metal-promoted catalysts (Co/HZSM-5 and Ni/HZSM-5) increased aromatic hydrocarbon yields and decreased the oxygenated compounds.

A pilot-scale bubbling fluidized bed reactor also enabled us to evaluate the products from biomass pyrolysis. This reactor operates based on the passage of the inert fluidization gas through a bed consisting of a mixture of sand and biomass, at a superficial velocity higher than the minimum fluidization. In this way, the biomass particles are rapidly heated, as they are mixed with the particles of sand previously heated by an external heating system. The vapors and biochar generated during the thermal degradation of biomass are removed from the reactor by the fluidized gas. Subsequently, these condensable vapors generate bio-oil. Pyrolysis in bubbling fluidized bed reactors provides good performance and a high yield of liquid products due to the short exposure time to low temperatures.^14,18^

Thus, this study aimed to evaluate the potential use of raw and torrefied Kraft lignin from paper and cellulose production in catalytic pyrolysis to generate value-added aromatic hydrocarbons for the chemical industry. We evaluated the effect of the HZSM-5 zeolite and HY-340 niobic acid catalysts. We also used analytical pyrolysis and an experimental unit for fast pyrolysis in a fluidized bed reactor to analyze the deoxygenation of the pyrolysis vapors from the kraft lignin torrefaction process.

## 2 Material and Methods

### 2.1 Material

In this study, we used industrial Kraft lignin (pH 4.05 in a 10% aqueous suspension) from Eucalyptus sp. supplied by a Brazilian pulp and paper mill. The Kraft lignin was produced from a black liquor containing approximately 40% solids. It was precipitated by acidification or pH reduction with the direct injection of CO2. Then, the solid–liquid phase was separated by using filtration. Finally, the filter cake was washed and dried. No additional preparation was required for Kraft lignin analysis. The physicochemical characterization of the Kraft lignin was performed in a previous study.^25^

The catalysts used in this work were the HZSM-5 zeolite manufactured by Alfa Aesar and the niobic acid HY-340 supplied by CBMM S/A. In the catalyst activation step, the HZSM-5 zeolite was calcined at 723 K for 6 h, and the HY-340 was calcined at 723 K for 2 h. After calcination, both samples were stored in a desiccator. The calcination temperature and time were chosen according to Santana Júnior et al.^21^ The physical properties of the catalysts are presented in previous literature.^16,21^

### 2.2 Torrefaction Pretreatment

The Kraft lignin was torrefied at 493, 533, and 573 K and named Lignin493, Lignin533, and Lignin573, respectively. In a previous work,^25^ Fourier transform infrared spectroscopy (FTIR) analyses effectively verified these torrefaction temperatures, showing structural changes compared with the raw biomass. Before pretreatment, kraft lignin samples were ground in a Willey knife mill (model SL-31) and classified using an ASTM sieve (Tyler series) with a 120 mesh size (Retsch brand) from which the passing particles were selected.

The torrefaction of Kraft lignin was carried out in an experimental unit designed by Silva.^26^ The experimental unit consisted of a muffle furnace (Linn Elektro Therm GMBH KK 260 SO 1060) with a temperature controller (N1200). For each torrefaction temperature, the capsule (reactor) was loaded with approximately 1.2 kg of Kraft lignin. The experiments were carried out at 2.5 K min^–1^, which is the maximum heating rate of the muffle furnace. At the beginning of the experiment, the muffle furnace was heated by electrical resistance from room temperature to torrefaction temperature. After the torrefaction temperature was reached, the system was kept at this temperature for 30 min. During torrefaction, some low molecular weight volatiles are released. Thus, the mass yields of the lignin samples by torrefaction temperature are 92.87, 89.25, and 82.72% for Lignin493, Lignin533, and Lignin573, respectively.

### 2.3 Analytical Pyrolysis Coupled to GC/MS (Py-GC/MS)

The analytical pyrolysis of raw and torrefied Kraft lignin was performed in a micropyrolyzer (CDS Analytical Pyroprobe 5200) coupled to a gas chromatograph-mass spectrometer (Shimadzu GC-MS QP 2010 plus). This system consisted of a quartz capillary reactor with a diameter of 2 mm and a length of 25 mm placed near the platinum resistance. For each experimental condition in the analytical pyrolysis experiments, 1 mg of the Kraft lignin sample was inserted into the capillary reactor. Two high-purity quartz wool (inert material) layers were used to maintain the sample in the center of the reactor and prevent it from moving along with the carrier gas during the analysis.

The analytical pyrolysis experiments of the raw and torrefied Kraft lignin were performed at 723, 823, and 923 K, which are temperatures commonly used in studies on biomass pyrolysis.^21,27–30^ After the final pyrolysis temperature was reached, the resistance remained heated for 10 s. The micropyrolyzer interface was kept at 348 K on standby and heated to 573 K for 1 min during pyrolysis. The pyrolyzer/GC transfer line and the pyrolyzer valve remained at 553 K.

The pyrolysis vapors from analytical pyrolysis were analyzed by using gas chromatography coupled to a mass spectrometer. The chromatograph used an Rtx-1701 capillary column, with 60 m length, 0.25 mm diameter, and 0.25 μm film thickness, to separate the compounds in the vapor phase. The carrier gas was helium (99.999% purity) at a flow rate of 1 mL min^–1^. The pyrolysis vapors were injected into the chromatograph after being split in a ratio of 1:90. In the gas chromatograph oven, a heating ramp consisted of an initial temperature of 318 K for 4 min, increasing to 553 K at 3 K min^–1^. During the experiments, we maintained the injector temperature at 523 K and the temperature of the chromatograph interface with the spectrometer at 548 K. The mass spectrometer has an electron impact ionization source set to operate at 473 K.

After analysis, the residues were removed from the capillary reactor. The micropyrolyzer was cleaned by using the clean function at 1273 K for 5 s. After every three analytical pyrolysis experiments, we conducted a blank analysis with the capillary reactor empty, i.e., without biomass, to clean the micropyrolyzer interface, micropyrolyzer/GC transfer line, and separation column. The data were processed using the National Institute of Standards and Technology (NIST, version 17), with a similarity index (SI) equal to or greater than 80%. Analyses were performed in triplicate for each experimental condition.

### 2.4 Catalytic Analytical Pyrolysis

In the catalytic analytical pyrolysis experiments, the raw Kraft lignin samples were mixed with HZSM-5 zeolite and HY-340 niobic acid, separately, in catalyst/biomass ratios of 1:1, 5:1, and 10:1. The homogeneity of the catalyst/biomass mixture can be evidenced by the consistent reproducibility of the results. The catalytic analytical pyrolysis procedure was the same as that described in Section 2.3.

Many studies have used high catalyst concentrations. Simão et al.,^31^ Santana Júnior et al.,^21^ and Chagas et al.^32^ performed catalytic pyrolysis at catalyst/biomass ratios of 1:1, 5:1, and 10:1. Wang and Brown^33^ performed experiments at catalyst/biomass ratios of 5:1, 10:1, and 20:1. In the catalyst activation step, the HZSM-5 zeolite was calcined at 723 K for 6 h, and the HY-340 niobic acid was calcined at 723 K for 2 h. After calcination, both catalysts were stored in a desiccator. The calcination temperature and time were selected based on Santana Júnior et al.^21^

### 2.5 Factorial Experimental Design (3^k^)

We performed a factorial experimental design of Lignin493, Lignin533, and Lignin573 samples using HZSM-5 zeolite and HY-340 niobic acid catalysts to determine the effect of the pyrolysis temperature and catalyst/biomass ratio on the production of aromatic hydrocarbons and deoxygenation of pyrolysis vapors from the torrefied Kraft lignin. The factorial experimental design identifies the effect of one or more experimental factors (input variables) on the responses (output variables) of a system under study. It uses statistical principles to obtain maximum information with the smallest possible number of experiments.^34^

In the factorial experimental design, factors are selected at different levels, and experiments are conducted for all combinations of the selected levels. The factorial experimental design can be represented by bk, where k is the number of factors and b is the number of levels.^35–37^ In the three-level factorial experimental design, the independent variables are coded as −1, 0, and 1, representing the lowest, central, and highest levels, respectively. Table 1 shows the factorial experimental design performed in this study.

**Table 1:** Factorial Experimental Design

exp.	*x*_1_	*x*_2_	exp.	*x*_1_	*x*_2_	exp.	*x*_1_	*x*_2_
1	–1	–1	4	–1	0	7	–1	+1
2	0	–1	5	0	0	8	0	+1
3	+1	–1	6	+1	0	9	+1	+1

The independent variables for the catalytic pyrolysis experiments of torrefied Kraft lignin were pyrolysis temperature (TP) and catalyst/biomass ratio (C), defined as *x*1 and *x*2, respectively. The response variable was the percentage of aromatic hydrocarbons (%HCA). The factorial experimental design of torrefied Kraft lignin was performed in duplicate. Table 2 shows the coded values of the independent variables. For Chen et al.,^38^ the torrefaction reduces oxygen content. Liang et al.^39^ and Vamvuka et al.^40^ reported that thermal degradation of lignin occurs gradually over a temperature range between 473 and 1123 K. Thus, the torrefaction temperatures selected can improve the characteristics of lignin fed into the reactor and reduce the oxygen content. This enables us to reduce the catalyst/biomass ratios to 1, 3, and 5.

**Table 2:** Independent Variables and the Corresponding Codification

level	*T*_P_(K)	*C* (mg catalyst/mg biomass)
–1	723	1
0	823	3
1	923	5

Eqs 1 and 2 are the coded equations for the independent variables. Eq 3 correlates the response *y* with the operational variables *x*_*i*_, considering the individual and quadratic effects of the independent variables, their interactions, and the associated adjustment error (ε). We can estimate the parameters β_*i*_, β*ii*, and β_*ij*_ in eq 3 based on the experimental values and the least-squares method. Multiple Regression Technique was used to determine the coded variables *x*_1_ (pyrolysis temperature) and *x*_2_ (catalyst/biomass ratio). The results of the factorial experimental design were evaluated using analysis of variance in the STATISTICA software, with a 95% confidence interval (*p* < 0.05).

$$x_{1}=\frac{T_{P}-823}{100}$$ 1

$$x_{2}=\frac{C-3}{2}$$ 2

$$y=\beta_{0}+\sum_{i=1}^{k}\beta_{i}x_{i}+\sum_{i=1}^{k}\beta_{ii}\overset{2}{\underset{i}{x}}+\sum_{i=1}^{k}\sum_{j=1}^{k}\beta_{i}x_{i}x_{j}+\varepsilon i<j$$ 3

### 2.6 Fast Pyrolysis in the Bubbling Fluidized Bed Reactor

Fast pyrolysis in a bubbling fluidized bed reactor was conducted to characterize the bio-oil by identifying the compounds through GC/MS and comparing them with the analytical pyrolysis results. As lignin is an amorphous polymer that behaves like a thermoplastic material, in the glass transition temperature range (*T*_g_), the sample expands and appears to be similar to rubber. Therefore, we performed fast pyrolysis in a bubbling fluidized bed reactor using Lignin573. This lignin sample was selected because it was outside the lignin expansion range, as reported by Moustaquim et al.,^41^ avoiding issues when feeding the sample into the reactor.

Figure 1 shows the experimental apparatus for fast pyrolysis in a fluidized bed reactor. It includes (1) speed transducer, (2) helical feed system, (3) fluidized bed reactor, (4) instrumentation and temperature control panel, (5) thermocouples, (6) cyclone, (7) thermostatic bath, (8) high voltage source, (9) electrostatic precipitator, and (10) an inert gas cylinder (nitrogen). The dimensions and characterization of these components are detailed in Oliveira,^28^ Carvalho,^29^ and Silva.^30^

**Figure 1:**
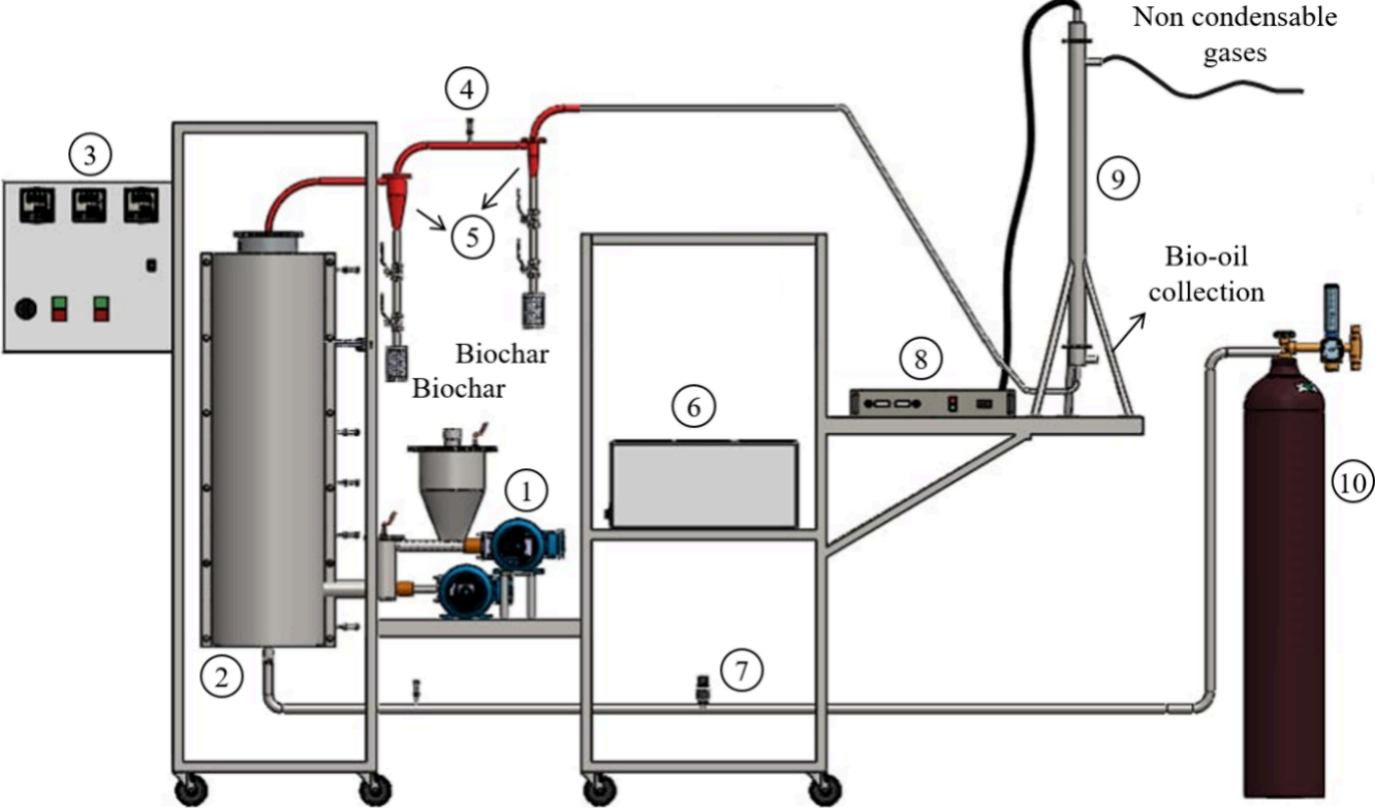
Experimental apparatus for fast pyrolysis in a bubbling fluidized bed reactor.

The experimental procedure initially consisted of loading the reactor with approximately 800 g of inert solid, sand no. 50, ranging between 0.3–0.6 mm, to guarantee the homogeneity of the mixture and high heat transfer coefficients. Then, the reactor was heated to 823 K, which is a typical temperature for fast pyrolysis. Upon reaching the set-point temperature, we started the fluidization of the bed using industrial nitrogen (inert gas) at a surface velocity of 0.22 m/s, 20% above the minimum fluidization velocity. Under these conditions, the minimum fluidization velocity was 0.18 m/s, as determined by Oliveira.^28^ Then, Lignin573 was fed by activating the helical screws to dose the biomass loaded into the reactor.

Cyclones separated the biochar produced during the pyrolysis. The aerosols and droplets formed during the cooling of condensable vapors were captured in the electrostatic precipitator with a voltage of 20 kV. The bio-oil collected (homogeneous liquid product) was stored in a bottle with a lid and refrigerated (273–277 K) until analysis. We analyzed the water content, viscosity, and pH of bio-oil according to ASTM E203-16, ASTM D445, and ASTME70-07, respectively. Analyses were performed in triplicate.

Furthermore, the compounds in the bio-oil were determined with a gas chromatography-mass spectrometer (GC/MS-QP2010 Plus Shimadzu). The bio-oil was diluted in high-purity chromatographic methanol (≥99.9%) and filtered through a syringe filter (PVDF 0.22 μm) to remove biochar particles that were not collected in the cyclones. The dilution consisted of approximately 0.5 mL (10 drops) of bio-oil in 10 mL of chromatographic methanol, i.e., 1/20. Around 1.8 mL of the diluted bio-oil was transferred to a 2 mL vial and injected into the chromatograph.

The chromatograph used a medium polarity Rtx-1701 capillary column (60 m × 0.25 mm × 0.25 μm) with a fused silica stationary phase (Restek). The carrier gas was helium (99.999% purity) at a flow rate of 1.08 mL min^–1^. During the analyses, the injector temperature remained at 523 K, the interface temperature at 548 K, and the ionization temperature at 473 K. The split ratio was 1:30. The chromatograph oven heating ramp had an initial temperature of 318 K, increasing to 553 K at 3 K min^–1^. We identified the compounds using the NIST library (version 17), with a similarity index greater than 80%.

## 3 Results and Discussion

### 3.1 Analytical Pyrolysis

We performed the analytical pyrolysis of raw and torrefied Kraft lignin at 723, 823, and 923 K. The analytical pyrolysis of torrefied Kraft lignin evaluated whether the torrefaction process changes the behavior of the generated compounds.

#### 3.1.1 Analytical Pyrolysis of Raw Kraft Lignin

The chromatographic peak area of a compound is directly proportional to its amount, and the percentage of the chromatographic peak area corresponds linearly to its content.^42^ Thus, the percentages of the chromatographic peak area can indicate changes in the composition of the pyrolysis vapors. A quantitative analysis could be performed by calibrating the equipment using analytical standards.^21^ However, this study focused on qualitative analysis of the pyrolysis vapors to evaluate selectivity by varying the experimental conditions. Figure 2 presents the means and standard deviations for the percentage of the chromatographic peak area for each group of compounds.

**Figure 2:**
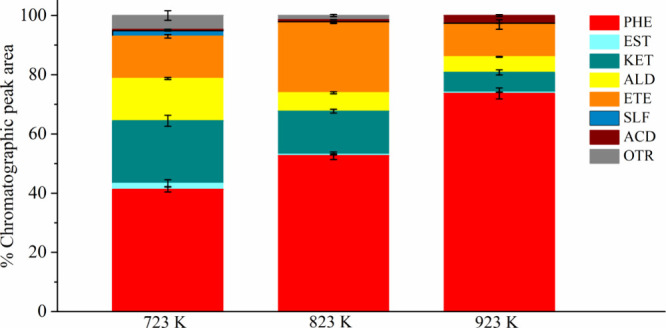
Analytical pyrolysis of raw Kraft lignin. Phenols (PHE), esters (EST), ketones (KET), aldehydes (ALD), ethers (ETE), sulfonates (SLF), acids (ACD), and others (OTR).

Figure 2 shows that the pyrolysis vapors from the analytical pyrolysis of raw Kraft lignin are a complex mixture of several compounds belonging to different groups, formed mainly by oxygenated compounds. The phenolic compounds presented the highest percentage of chromatographic peak area. We can observe that the increase in the pyrolysis temperature increased the production of phenolic compounds. There was no production of hydrocarbons at any of the temperatures studied. The amount of phenolic compounds increased by approximately 78% by increasing the pyrolysis temperature from 723 to 923 K. Thus, the production of phenolic compounds reached a maximum of 74% of the chromatographic peak area at 923 K. In contrast, the production of ketones and aldehydes decreased with increasing temperature.

Santana Júnior et al.^21^ reported similar results. According to Lund et al.,^43^ the pyrolysis temperature strongly influences the amount of products since an increase in the pyrolysis temperature will increase the amount of some products up to a maximum temperature. Zhao et al.^44^ noted that the production of phenolic compounds increased by increasing Py-GC/MS temperature due to increased cleavage of the methoxyl group in the aromatic rings through demethoxylation, demethylation, and dehydroxylation reactions. Jiang et al.^45^ performed micropyrolysis of Alcell lignin and Asian lignin, and they identified and quantified the compounds for each lignin in a temperature range of 673–1073 K. The authors found that the maximum yield of phenolic compounds was obtained at 873 K for both lignins.

The production of phenolic compounds from the pyrolysis of raw Kraft lignin is important for the chemical industry due to the diverse applications of these compounds, which add value to this biomass. Phenolic compounds can produce resins, adhesives, and polymers, act as intermediates in syntheses for the pharmaceutical industry, and serve as flavorings in the food industry.^46,47^ However, phenolic compounds are undesirable for producing bio-oil for fuel purposes due to instability caused by oxidation reactions. These compounds can be separated from the bio-oil through distillation or extraction.^32^ Table 3 presents the main compounds generated in the analytical pyrolysis of raw Kraft lignin based on the percentage of the chromatographic peak area for the temperatures studied.

**Table 3:** Main Compounds Generated in the Analytical Pyrolysis of Raw Kraft Lignin

compounds	formula	723 K	823 K	923 K
2-methoxyphenol (guaiacol)	C_7_H_8_O_3_	2.69 ± 0.35	4.96 ± 0.31	4.04 ± 0.15
2-methoxy-4-methylphenol (creosol)	C_8_H_10_O_2_	1.86 ± 0.15	5.49 ± 0.15	3.56 ± 0.24
1,2-benzenediol,3-methoxy	C_7_H_8_O_3_		7.72 ± 0.43	10.22 ± 0.08
2,6-dimethoxyphenol (syringol)	C_8_H_10_O_3_	14.80 ± 0.96	20.40 ± 0.72	11.11 ± 1.18
1,2,4-trimethoxybenzene	C_9_H_12_O_3_	6.68 ± 0.54	15.09 ± 0.11	6.22 ± 0.99
1,2,3-trimethoxy-5-methyl-benzene	C_10_H_14_O_3_	6.37 ± 0.16	6.97 ± 0.34	2.43 ± 0.70
2,6-dimethoxy-4-(2-propenyl)-phenol	C_11_H_14_O_3_	10.95 ± 0.33	1.80 ± 0.08	1.76 ± 0.34
4-hydroxy-3,5-dimethoxybenzaldehyde (syringaldehyde)	C_9_H_10_O_4_	8.85 ± 0.20	3.86 ± 0.17	2.43 ± 0.22
1-(4-hydroxy-3,5-dimethoxyphenyl)-ethanone	C_10_H_12_O_4_	11.05 ± 0.49	6.58 ± 0.36	2.60 ± 0.27

Table 3 shows that 2,6-methoxyphenyl (syringol), which belongs to the phenolic group, was the main compound formed in the pyrolysis of raw Kraft lignin at the three temperatures. In this group, guaiacol and creosol were also formed. The main compounds formed in the ketone, ether, and aldehyde groups were 1-(4-hydroxy-3,5-dimethoxyphenyl)-ethanone, 1,2,4-trimethoxybenzene, and 4-hydroxy-3,5-dimethoxybenzaldehyde (syringaldehyde), respectively. According to Dong et al.,^48^ lignin can undergo demethoxylation, demethylation, and alkylation reactions. Demethoxylation reactions can form compounds, such as phenol, guaiacol, and others. Demethylation and alkylation reactions can generate catechol and cresol.

The content of creosol, guaiacol, and syringol increased with increasing temperature from 723 to 823 K. However, the content of these compounds decreased by increasing temperature from 823 to 923 K. Similarly, Chen et al.^49^ performed micropyrolysis of lignin and observed that the contents of cresol, guaiacol, and syringol decreased by increasing the temperature from 773 to 873 K. Shen et al.^50^ performed Py-GC/MS of rice straw, rice husk, and maple lignin extracted by the Klason method at 823, 923, 1073, and 1173 K. They found that syringol and guaiacol-type compounds were predominant in the pyrolysis of maple lignin at all temperatures, while these compounds were predominant in the pyrolysis of rice straw and rice husk at 823 and 923 K.

Syringol is a compound of significant interest to the food industry since it is responsible for the smoky aroma of foods. Guaiacol and its derivatives are used in the pharmaceutical industry, due to their expectorant, antiseptic, and local anesthetic properties, and in the food industry, as flavoring agents.^19^ The 4-hydroxy-3,5-dimethoxybenzaldehyde is a value-added compound due to its properties in medicinal chemistry. This compound has emerged as a promising drug in the fight against colon cancer. In addition, it has anti-inflammatory effects.^51^ For fuel applications, reducing oxygenated compounds in bio-oil is essential. One of the techniques for using bio-oil as ignition fuel in transportation or boilers is to produce an emulsion with other fuel sources, such as diesel or biodiesel, using surfactants.^52^ However, the oxygenated compounds in bio-oil add polarity, which promotes miscibility, making it difficult to mix with petroleum-derived fuels.

#### 3.1.2 Analytical Pyrolysis of Torrefied Kraft Lignin

We performed the analytical pyrolysis of torrefied Lignin493, Lignin 533, and Lignin573 samples at 723, 823, and 923 K. Figure 3 shows the means and standard deviations for the percentage of the chromatographic peak area for each group of compounds.

**Figure 3:**
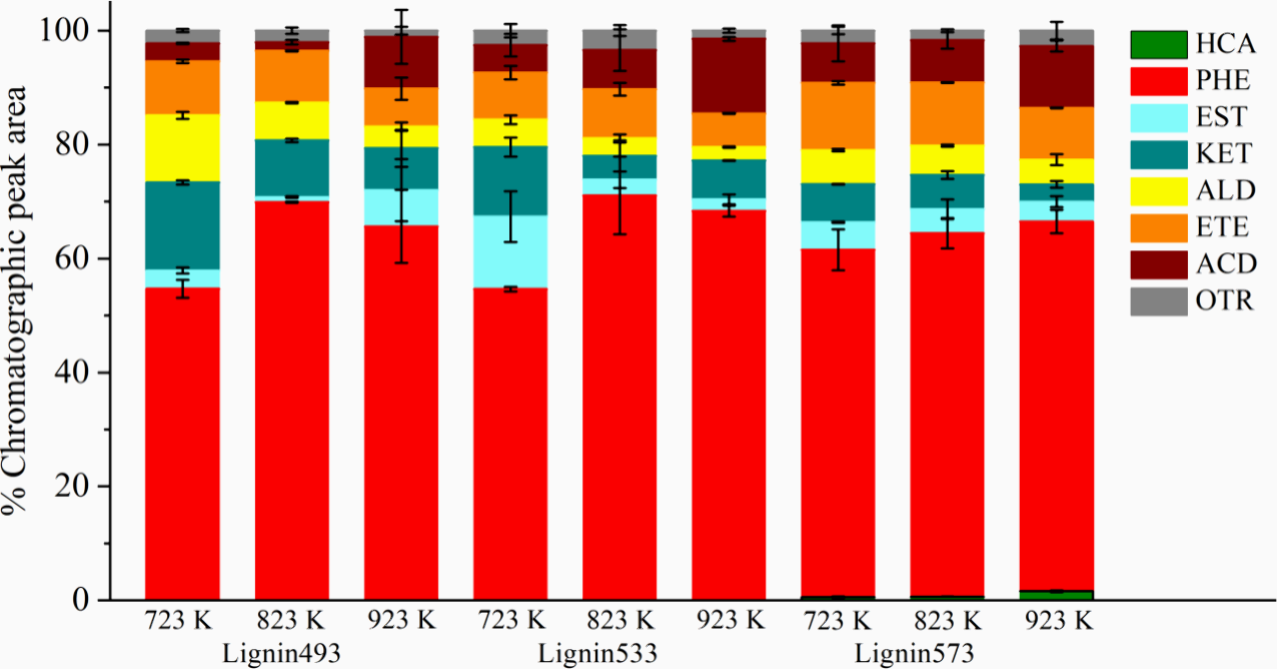
Analytical pyrolysis of torrefied Kraft lignin samples. Aromatic hydrocarbons (HCA), phenols (PHE), esters (EST), ketones (KET), aldehydes (ALD), ethers (ETE), sulfonated (SLF), acids (ACD), and others (OTR).

Figure 3 shows that phenolic compounds presented the highest percentages of the chromatographic peak area at all three temperatures studied. The production of phenolic compounds is primarily attributed to the cleavage of the ether bond and the demethoxylation reaction of lignin, which alters its structure.^53^ Similarly to the analytical pyrolysis of raw Kraft lignin, 2,6-dimethoxyphenol (syringol) was the main compound formed under all experimental conditions. For Lignin573 samples, aromatic hydrocarbons are produced at all three pyrolysis temperatures studied. However, this did not occur for Lignin493 and Lignin533. Since the temperature range of thermal degradation of lignin is wide, the torrefaction significantly changes product distribution. After torrefaction, most of the C–C bonds within and between the alkyl chains in lignin become unstable and reactive, causing further fragmentation to release −H, −OH, −CH3, and −COOH during pyrolysis. Thus, torrefaction at 573 K significantly influenced the analytical pyrolysis of Kraft lignin.^49^

The raw and torrefied lignin samples (Lignin493, Lignin533, and Lignin573) were characterized by X-ray diffraction (XRD), scanning electron microscopy (SEM), energy-dispersive X-ray fluorescence (EDXRF) spectrometry, thermogravimetry, proximate and elemental analysis in a previous work of the research group.^25^ It was found that the raw and torrefied lignin samples have similar characteristics, and the increase in the torrefaction temperature resulted in a reduction in the O/C and H/C ratios of the biomass, which is desirable for the pyrolysis of the material.

Ren et al.^54^ studied the effect of torrefaction on the composition of sawdust bio-oil produced via microwaves. The authors noted that torrefaction favored the production of phenols and sugars compared with the pyrolysis of raw biomass. They also observed the production of hydrocarbons (between 3.21 and 7.50% chromatographic peak area) and the reduction of organic acids. Mahadevan et al.^55^ performed analytical pyrolysis of lignin samples extracted from southern pine and switchgrass via organosolv extraction at 773 K, both in their raw and torrefied forms (423, 448, 473, and 498 K). They identified aromatic hydrocarbons, phenols, guaiacols, and syringols. They also found that increasing the torrefaction temperature significantly decreased the total yield of guaiacols, increased the yield of phenols, and did not produce hydrocarbons for any of the samples. Zhang et al.^53^ studied the analytical pyrolysis of torrefied apricot shell lignin at 473, 513, and 553 K. They observed that as the torrefaction temperature increased, the content of phenolic compounds initially increased and then slightly decreased.

### 3.2 Effect of Temperature and Catalysts on the Pyrolysis of Raw Kraft Lignin

#### 3.2.1 HZSM-5 Zeolite

We performed catalytic analytical pyrolysis to evaluate the deoxygenation of pyrolysis vapors from raw Kraft lignin and the production of aromatic hydrocarbons. We analyzed the effects of HZSM-5 zeolite on the catalyst/biomass ratios (1:1, 5:1, and 10:1) and the increase in the pyrolysis temperature (723, 823, and 923 K). Figure 4 shows the means and standard deviations for the percentages of the chromatographic peak area for each group of compounds.

**Figure 4:**
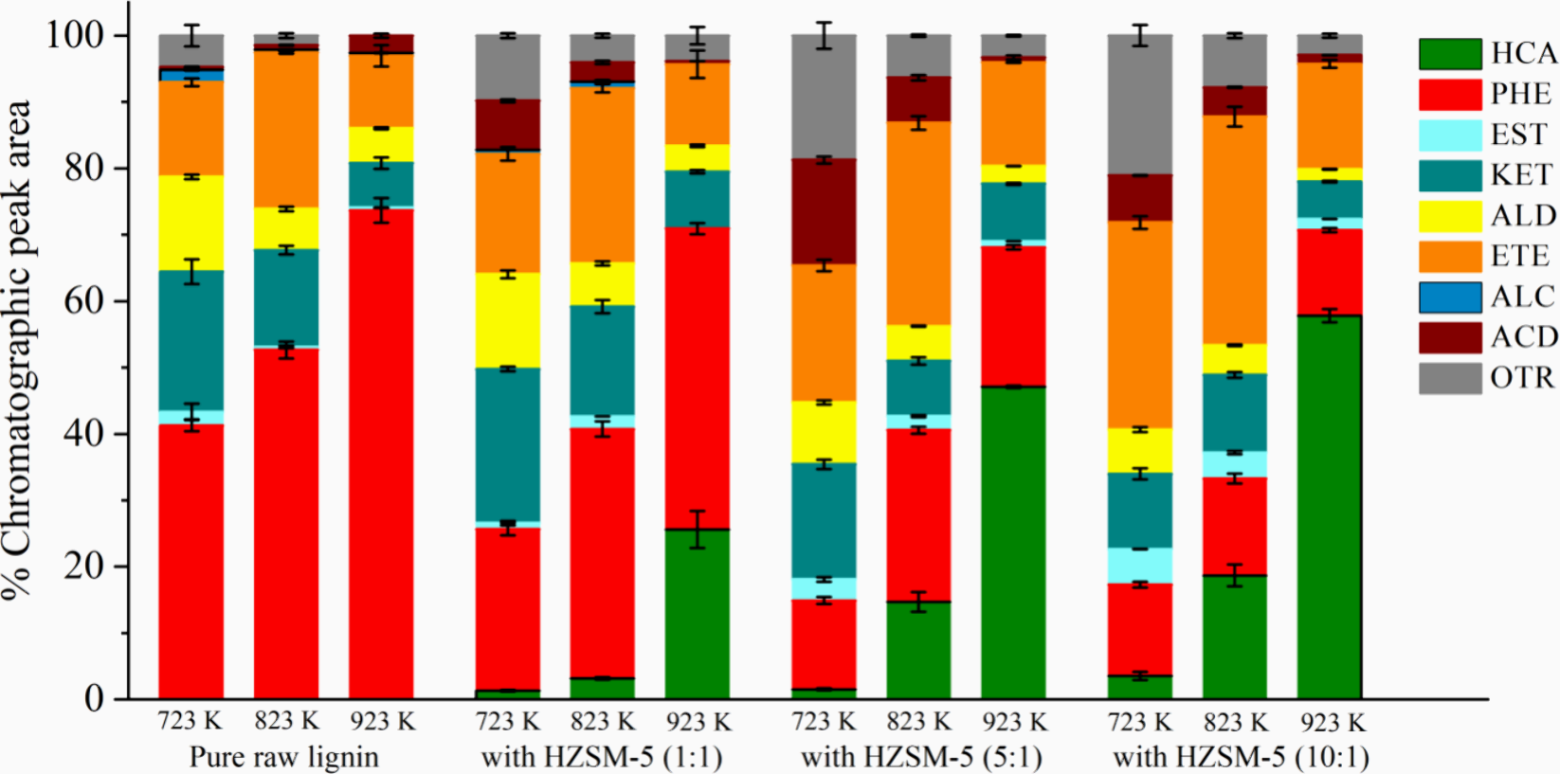
Catalytic analytical pyrolysis of raw Kraft lignin with a HZSM-5 zeolite. Hydrocarbons (HCA), phenols (PHE), esters (EST), ketones (KET), aldehydes (ALD), ethers (ETE), alcohols (ALC), acids (ACD), and others (OTR).

Figure 4 shows that catalytic analytical pyrolysis produced aromatic hydrocarbons. The production of aromatic hydrocarbons increased by increasing the catalyst/biomass ratio and pyrolysis temperature. The catalyst/biomass ratio of 10:1 and the pyrolysis temperature at 923 K led to the highest selectivity for aromatic hydrocarbons. At 723 K, regardless of the catalyst/biomass ratio, we can observe a slight increase in the production of aromatic hydrocarbons compared to noncatalytic pyrolysis. At 823 K, the catalyst/biomass ratio of 1:1 slightly increased the production of aromatic hydrocarbons. However, the catalyst/biomass ratio of 5:1 and 10:1 showed significant production of aromatic hydrocarbons, reaching a maximum of 18.69% at 10:1. At 923 K, the selectivity for aromatic hydrocarbons increased considerably, regardless of the catalyst/biomass ratio, reaching a maximum of 57.84% at 10:1.

HZSM-5 zeolite can promote the dissociation, deoxidation, decarboxylation, decarbonylation, and deoxygenation reactions of oxygenated compounds in pyrolysis vapors. This reduces their content due to the strong acidity and the large number of acid sites in the catalyst, which favor the production of aromatic compounds by aromatization and isomerization reactions. In addition, the complex porous structure of zeolites also plays an important role in the thermal conversion process and hydrocarbon selectivity.^56,57^

Since the HZSM-5 zeolite was efficient in the selectivity for aromatic hydrocarbons (see Figure 3), the phenolic compounds and ketones decreased by increasing the catalyst/biomass ratio. This reduced the oxygenated compounds in the pyrolysis of raw Kraft lignin. Thus, the production of phenolic compounds was 74% at 923 K in the analytical pyrolysis of pure raw lignin (Figure 2) and 45.34% at 923 K with a catalyst/biomass ratio of 1:1 (Figure 4), reducing to 13.72% (average between the three temperatures) with a catalyst/biomass ratio of 10:1 (Figure 4). The production of ketones was 23.21% at 723 K, 16.53% at 823 K, and 8.56% at 923 K with a catalyst/biomass ratio of 1:1 and reduced to 11.34% at 723 K, 11.70% at 823 K, and 5.62% at 923 K with a catalyst/biomass ratio of 10:1 (Figure 4). The deoxygenation of phenolic compounds and the production of aromatic hydrocarbons may be attributed to their diffusion into the zeolite pores.^38^

HZSM-5 zeolite increased selectivity for xylene (18.16%), toluene (11.22%), 1-methylnaphthalene (10.50%), benzene (7.35%), naphthalene (5.42%), and others by reducing 2,6-dimethoxyphenol (syringol), 3-methoxy-1,2-benzenediol, 2-methoxy-4-methylphenol (creosol), and 2-methoxyphenol (guaiacol). According to Santana Júnior,^21^ the aromatic hydrocarbons formed during the catalytic pyrolysis of Kraft lignin using HZSM-5 zeolite can be used in gasoline blending, solvents, or raw materials for various high-value-added chemicals.

Benzene is primarily used as a raw material to produce ethylbenzene (transformed into styrene) and cumene (a precursor for phenol production). It is also used to synthesize cyclohexane, a precursor for nylon production. Toluene is used in refinery streams for gasoline blending to improve the octane rating. Xylene is widely used in plastic bottles and polyester clothing, as well as in solvents for applications ranging from cleaning circuit boards to diluting paints and varnishes. Xylene can also be used in refinery streams for gasoline blending or separated into isomers for chemical applications.^58^ Mullen and Boateng^59^ performed catalytic pyrolysis of lignin from four different sources using HZSM-5 zeolite in the Py-GC/MS system at 923 K. The authors found that the selectivity for aromatic hydrocarbons varied with the lignin composition, and the main route to produce hydrocarbons was probably the increase in the depolymerization efficiency that releases and converts the aliphatic ligands of lignin, followed by aromatization.

Ohra-Aho and Linnekoski^60^ studied the catalytic pyrolysis of Kraft lignin and Scots pine wood at 873 K using HZSM-5, Y, and Pd/C zeolites at a catalyst/biomass ratio of 1:1. The authors observed an increase in the production of aromatic hydrocarbons using zeolites when compared to noncatalytic pyrolysis. They also noted that Kraft lignin produced lower hydrocarbons than Scots pine wood. However, all catalysts could promote the deoxygenation of the pyrolysis vapors. Chen et al.^61^ performed catalytic pyrolysis of sawdust and sorghum residue at 773 K using HZSM-5 zeolite at catalyst/biomass ratios of 0:1, 1:1, 5:1, and 10:1. The yield of aromatic hydrocarbons increased by adding the catalyst, reaching a maximum of 32.95% in the pyrolysis of sawdust and 43.70% in the pyrolysis of the sorghum residue.

#### 3.2.2 HY-340 Niobic Acid

In the literature, few studies have employed HY-340 niobic acid. It can be a good catalyst in reactions involving water molecules due to its high acidity and strong metal–support interaction, which is necessary for active and stable catalysts, and it can also be used in pyrolysis processes.^31^ We used HY-340 niobic acid in the catalytic analytical pyrolysis of raw Kraft lignin to evaluate the deoxygenation of pyrolysis vapors and the production of aromatic hydrocarbons. Similarly to HZSM-5 zeolite, we analyzed the effects of HY-340 niobic acid on the catalyst/biomass ratios (1:1, 5:1, and 10:1) and the increase in the pyrolysis temperature (723, 823, and 923 K). Figure 5 presents the means and standard deviations for the percentage of the chromatographic peak area for each group of compounds.

**Figure 5:**
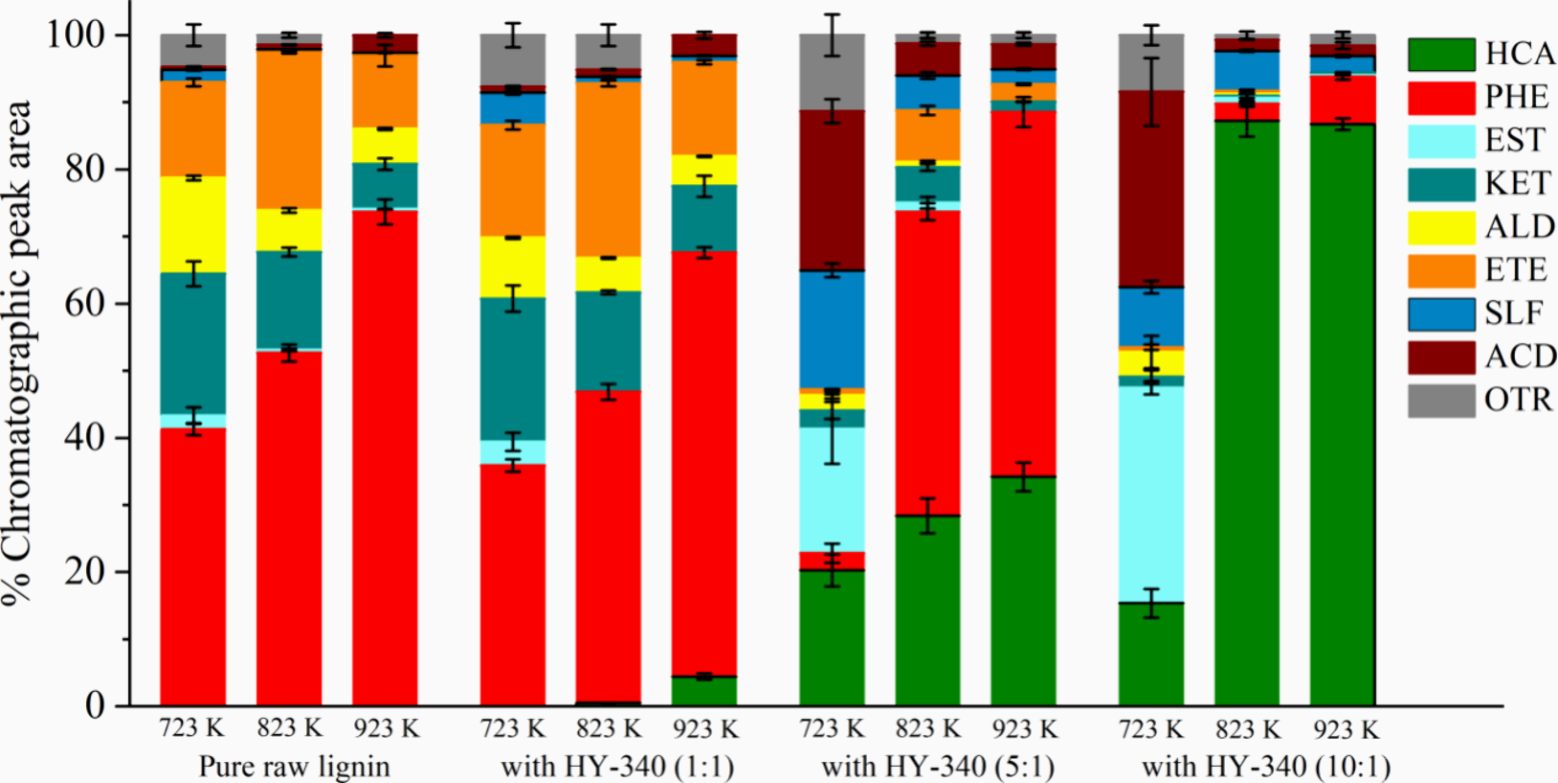
Catalytic analytical pyrolysis of raw Kraft lignin with HY-340 niobic acid. Hydrocarbons (HCA), phenols (PHE), esters (EST), ketones (KET), aldehydes (ALD), ethers (ETE), sulfonated (SLF), acids (ACD), and others (OTR).

Figure 5 shows the effect of HY-340 niobic acid and the temperature on the catalytic pyrolysis of raw Kraft lignin. The production of aromatic hydrocarbons increased by increasing the catalyst/biomass ratio and the pyrolysis temperature. The highest selectivity for aromatic hydrocarbons is achieved at 10:1 and 823 and 923 K. For the catalyst/biomass ratio of 1:1, there was no production of aromatic hydrocarbons at 723 and 823 K. However, at 923 K, the production of aromatic hydrocarbons slightly increased compared to noncatalytic pyrolysis, reaching only 4.43%. For the catalyst/biomass ratio of 5:1, the production of aromatic hydrocarbons increased by increasing pyrolysis temperature, with a selectivity of 20.25% at 723 K, 28.37% at 823 K, and 34.20% at 923 K. For the catalyst/biomass ratio of 10:1, the selectivity for aromatic hydrocarbons at 723 K was lower than in the 5:1 condition. The selectivity for aromatic hydrocarbons was significant at 823 and 923 K, producing 87.24 and 86.75% of aromatic hydrocarbons, respectively. Thus, maximum selectivity can be obtained at 723 K, reducing operating costs compared to 923 K.

HY-340 niobic acid provides high catalytic activity in the hydrodeoxygenation and deoxygenation of lignin due to its acidic properties, particularly its Lewis acid sites and mesoporous structure, which can restrict repolymerization reactions and promote molecular diffusion.^62^ It decreases the production of oxygenated compounds by increasing the catalyst/biomass ratios. The good performance of this catalyst is associated with the ability of a transition metal oxide to break the C–O bond and act as a solid acid catalyst for dehydration. Niobium-containing oxides show high activity, selectivity, and stability for acid catalysis, especially for hydrogenolysis, dehydration, and hydrodeoxygenation reactions.^15^

The increase in the catalyst/biomass ratio, together with the increase in the pyrolysis temperature, increased selectivity for benzene and its derivatives (toluene, ethylbenzene, xylene, and others) by decreasing 2,6-dimethoxyphenol (syringol), 3-methoxy-1,2-benzenediol, 2-methoxy-4-methylphenol (creosol), and 2-methoxyphenol (guaiacol). BTEX compounds (benzene, toluene, ethylbenzene, and xylene) are valuable and of high commercial interest, and they can be obtained by pyrolysis. They are widely used to produce paints and varnishes, adhesives, thinners, inks, rubber products, cosmetics, pharmaceuticals, and other chemical and petrochemical products. In addition, lighter hydrocarbons have greater volatility, which is suitable for commercial applications as fuels.^63^ In experiments using HY-340 niobic acid, we also observed the formation of sulfonated compounds, such as dimethyl disulfide (DMDS) and thiophene. DMDS is a volatile sulfuric acid used as a repellent, nematicide, and soil disinfectant.^64^

Carvalho et al.^15^ performed catalytic pyrolysis of sorghum bagasse using HY-340 niobic acid at catalyst/biomass ratios of 1:1, 2:1, 5:1, and 10:1 and 723, 823, and 923 K. The authors noted that the formation of furans increased at catalyst/biomass ratios of 1:1 and 2:1, and the oxygenated compounds decreased by increasing the catalyst/biomass ratios at all temperatures. They reported 54% content of olefins in the experiments with catalyst/biomass ratios of 2:1 and 5:1 at 923 K. Li et al.^62^ used HY-340 niobic acid in the catalytic pyrolysis of enzymatic hydrolysis lignin, at catalyst/biomass ratios of 1:1, 3:1, 5:1, 7:1, and 9:1 and a pyrolysis temperature range of 773–923 K. The yield of aromatic hydrocarbons and monocyclic aromatic hydrocarbons was 11.2 and 94%, respectively, by mass, at a catalyst/biomass ratio of 9:1 and 923 K.

It was found that the use of catalysts altered the distribution of compounds from the pyrolysis of Kraft lignin. In addition, the use of catalysts probably changes the production and contents of byproducts. Thus, future work can be carried out to evaluate potential emissions and waste handling methods for catalytic pyrolysis using HZSM-5 zeolite and HY-340 niobic acid.

### 3.3 Factorial Experimental Design

We evaluated the effect of the pyrolysis temperature and catalyst/biomass ratio on the deoxygenation of pyrolysis vapors from torrefied Kraft lignin and the production of aromatic hydrocarbons. We employed the 3^k^ factorial experimental design for torrefied lignin samples using HSZM-5 zeolite and HY-340 niobic acid catalysts. We used catalyst/biomass ratios of 1:1, 3:1, and 5:1 (mg of catalyst/mg of biomass). These values are lower than those employed in the catalytic analytical pyrolysis of raw Kraft lignin since the torrefaction reduced a small fraction of the oxygen content, and the deoxygenation of pyrolysis vapors can be obtained with lower catalyst/biomass ratios, reducing operating costs. We identified the compounds generated in the catalytic analytical pyrolysis of torrefied Kraft lignin and quantified the %HCA produced in each experimental condition. Tables 4 and 5 present the %HCA obtained in the factorial experimental design of the torrefied Kraft lignin using HZSM-5 zeolite and HY-340 niobic acid, respectively.

**Table 4:** Factorial Experimental Design for Torrefied Lignin Samples Using HZSM-5 Zeolite

			average %HCA		
exp	*x*_1_	*x*_2_	lignin493	lignin533	lignin573
1	–1	–1	2.65 ± 0.01	1.86 ± 0.76	1.77 ± 0.05
2	0	–1	18.32 ± 0.02	13.73 ± 0.26	23.29 ± 0.25
3	1	–1	32.42 ± 0.43	33.95 ± 0.18	43.89 ± 1.22
4	–1	0	1.98 ± 0.38	3.93 ± 0.15	44.09 ± 1.63
5	0	0	22.03 ± 0.17	25.62 ± 1.48	76.65 ± 0.62
6	1	0	41.36 ± 0.01	50.22 ± 2.69	73.12 ± 2.48
7	–1	1	5.55 ± 0.51	24.43 ± 1.09	66.78 ± 1.50
8	0	1	20.75 ± 1.76	34.90 ± 1.00	92.84 ± 0.65
9	1	1	41.95 ± 1.16	53.72 ± 1.58	87.95 ± 2.61

**Table 5:** Factorial Experimental Design for Torrefied Lignin Samples Using HY-340 Niobic Acid

			average %HCA		
exp	*x*_1_	*x*_2_	lignin493	lignin533	lignin573
1	–1	–1	1.33 ± 0.25	0.46 ± 0.08	3.41 ± 0.15
2	0	–1	3.09 ± 0.04	0.65 ± 0.10	5.38 ± 0.19
3	1	–1	4.71 ± 0.55	1.15 ± 0.08	5.75 ± 0.27
4	–1	0	7.34 ± 0.40	31.32 ± 0.34	55.86 ± 0.85
5	0	0	2.89 ± 0.23	7.31 ± 0.61	69.78 ± 1.15
6	1	0	4.47 ± 0.12	11.62 ± 0.30	58.67 ± 2.18
7	–1	1	29.29 ± 1.68	45.16 ± 3.08	78.78 ± 0.49
8	0	1	8.71 ± 0.68	50.02 ± 0.58	87.72 ± 1.38
9	1	1	19.91 ± 0.42	48.95 ± 1.67	90.02 ± 0.96

Table 4 shows that for Lignin493 and Lignin533, the maximum %HCA using HZSM-5 zeolite was 41.95 and 53.72%, respectively, at a pyrolysis temperature of 923 K and a catalyst/biomass ratio of 5:1 (experiment 9). For Lignin573, the maximum %HCA was 92.84% at a pyrolysis temperature of 823 K and a catalyst/biomass ratio of 5:1 (experiment 8). Analyzing Table 5, the maximum %HCA with HY-340 niobic acid was 29.29% (Lignin493, experiment 7), 50.02% (Lignin533, experiment 8), and 90.02% (Lignin573, experiment 9), corresponding to pyrolysis temperatures of 723, 823, and 923 K, respectively, and a catalyst/biomass ratio of 5:1.

In addition, the production of aromatic hydrocarbons increased with an increase in the torrefaction temperature. The selectivity for aromatic hydrocarbons increased from 41.95% in Lignin493 to 92.84% in Lignin573 when using HZSM-5 zeolite (Table 4) and from 29.29% in Lignin493 to 90.02% in Lignin573 when using HY-340 niobic acid (Table 5). Lignin573 achieved the highest selectivity for aromatic hydrocarbons with HZSM-5 zeolite (92.84%) and HY-340 niobic acid (90.02%) at a catalyst:biomass ratio of 5:1. These results were better than those achieved by raw Kraft lignin (Figures 3 and 4) using HZSM-5 zeolite (57.84%) and HY-340 niobic acid (87.24%) at a catalyst/biomass ratio of 10:1. Thus, these results show that the torrefaction process reduces the use of catalyst in catalytic analytical pyrolysis, providing maximum deoxygenation of pyrolysis vapors and better selectivity for aromatic hydrocarbons with a lower catalyst/biomass ratio than that of raw Kraft lignin, reducing operating costs.

In both factorial experimental designs, the %HCA increased by increasing the independent variables; i.e., they promoted the deoxygenation of pyrolysis vapors from the torrefied Kraft lignin. The experiments using both catalysts indicated that an increase in the catalyst/biomass ratio to 5 was essential to achieve maximum deoxygenation and the highest %HCA. In the literature, there is a lack of studies describing the use of HY-340 niobic acid together with torrefied biomass in the catalytic pyrolysis process.

For Chen et al.,^38^ the production of aromatic hydrocarbons can be increased by increasing torrefaction temperature and adding HZSM-5 zeolite. This indicates that the catalyst and torrefaction temperature promoted the deoxygenation of bio-oil to produce aromatic hydrocarbons. Adhikari et al.^65^ stated that the acidity of the HZSM-5 zeolite was highly favorable for producing aromatic hydrocarbons in the pyrolysis of lignin after torrefaction. They produced a large amount of aromatic hydrocarbons (∼35%) from the pyrolysis of torrefied lignin at 498 K using HZSM-5 zeolite, at a catalyst/biomass ratio of 4:1 and 873 K, showing that torrefaction favored high production of aromatic hydrocarbons in catalytic pyrolysis. Mahadevan et al.^55^ performed in situ catalytic analytical pyrolysis of fresh and torrefied (between 423 and 498 K) pine and switchgrass lignin at 773 K using an HZSM-5 zeolite ratio of 1:4. They observed that the torrefaction negatively affected the production of hydrocarbon compared to the pyrolysis of fresh lignin The yield of aromatic hydrocarbon significantly decreased by increasing torrefaction temperature, varying from 11.6 to 4.9% for pine lignin and from 10.4% to 7.1% for switchgrass lignin. However, Huang et al.^66^ showed that the pyrolysis of lignin is complex and depends on several factors, including the source of lignin and its physicochemical properties.

Considering Tables 4 and 5, we determined the parameters for eq 3. Eqs 4–6 and 7–9 represent the %HCA as a function of the independent variables, in coded form, at a 95% confidence level using HZSM-5 zeolite and HY-340 niobic acid for each torrefied lignin sample, respectively. The coefficients of determination (*R* ^2^) for eqs 4–9 were 0.98, 0.95, 0.98, 0.84, 0.92, and 0.99, respectively. Given the confidence level, some parameters of the empirical equations had no statistical significance. Thus, we presented only the significant parameters in the equations. Multiple regression tables with parameter values and significance levels (p-level) for the full and reduced models are provided in the Supporting Information.

$$\%{\mathrm{HCA}}_{\mathrm{HZSM}-5}^{\mathrm{lignin}493}(\%)=20.78+(17.592.78)\left(\frac{x_{1}}{x_{2}}\right)+{\left(\frac{x_{1}}{x_{2}}\right)}^{T}\left(\begin{array}{cc} 0 & 0.58\\ 0.58 & 0 \end{array}\right)\left(\frac{x_{1}}{x_{2}}\right)$$ 4

$$\%{\mathrm{HCA}}_{\mathrm{HZSM}-5}^{\mathrm{lignin}533}(\%)=26.93+(17.9510.58)\left(\frac{x_{1}}{x_{2}}\right)$$ 5

$$\%{\mathrm{HCA}}_{\mathrm{HZSM}-5}^{\mathrm{lignin}573}(\%)=72.17+(15.3929.77)\left(\frac{x_{1}}{x_{2}}\right)+{\left(\frac{x_{1}}{x_{2}}\right)}^{T}\left(\begin{array}{cc} -11.32 & -2.62\\ -2.62 & -11.87 \end{array}\right)\left(\frac{x_{1}}{x_{2}}\right)$$ 6

$$\%{\mathrm{HCA}}_{\mathrm{HY}-340}^{\mathrm{lignin}493}(\%)=8.12+{\left(\frac{x_{1}}{x_{2}}\right)}^{T}\left(\begin{array}{cc} 6.27 & -1.56\\ -1.56 & -6.28 \end{array}\right)\left(\frac{x_{1}}{x_{2}}\right)$$ 7

$$\%{\mathrm{HCA}}_{\mathrm{HY}-340}^{\mathrm{lignin}533}(\%)=16.57+(023.64)\left(\frac{x_{1}}{x_{2}}\right)+{\left(\frac{x_{1}}{x_{2}}\right)}^{T}\left(\begin{array}{cc} 0 & 0\\ 0 & 7.83 \end{array}\right)\left(\frac{x_{1}}{x_{2}}\right)$$ 8

$$\%{\mathrm{HCA}}_{\mathrm{HY}-340}^{\mathrm{lignin}573}(\%)=65.13+(2.7340.33)\left(\frac{x_{1}}{x_{2}}\right)+{\left(\frac{x_{1}}{x_{2}}\right)}^{T}\left(\begin{array}{cc} -5.54 & 0\\ 0 & -16.26 \end{array}\right)\left(\frac{x_{1}}{x_{2}}\right)$$ 9

We can observe that the torrefied Kraft lignin samples showed distinct behaviors since the equations did not present the same significant parameters within the 95% confidence interval. Therefore, the selectivity for aromatic hydrocarbons can vary based on the selection of torrefied lignin samples, pyrolysis temperatures, and catalysts. Some uncontrollable variables may be related to these results, such as the reaction mass (volatiles) under each experimental condition and the occurrence of secondary reactions.

For Lignin493 with HZSM-5 zeolite (eq 4), we can observe significant and positive linear effects of the independent variables and their interaction on %HCA. Under average operating conditions, the pyrolysis temperature (*x*_1_) strongly influenced the %HCA. The effect of the pyrolysis temperature was approximately 6.33 times higher than the catalyst/biomass ratio (*x*_2_) on the %HCA. However, for Lignin493 with HY-340 niobic acid (eq 7), we did not observe significant linear effects. The quadratic effects of the independent variables increased the %HCA, while the interaction between variables decreased the %HCA.

For Lignin533 with HZSM-5 zeolite (eq 5), we can observe significant and positive linear effects of the independent variables on %HCA. Considering average operating conditions, the pyrolysis temperature (*x*_1_) showed the highest influence on %HCA. The effect of the pyrolysis temperature (*x*_1_) was 1.70 times higher than the catalyst/biomass ratio (*x*_2_). For Lignin533 with HY-340 niobic acid (eq 8), the linear and quadratic effects of the catalyst/biomass ratio (*x*_2_) on %HCA were significant and positive.

For Lignin573 with HZSM-5 zeolite (eq 6), we can observe that the linear, quadratic, and interaction effects of the independent variables were significant on %HCA. The linear effects positively influenced %HCA, and the quadratic and interaction effects negatively affected %HCA. Considering average operating conditions, the catalyst/biomass ratio (*x*_2_) strongly influenced %HCA. Thus, the effect of the catalyst/biomass ratio was approximately 1.93 times higher than the pyrolysis temperature (*x*_1_) on %HCA. However, for Lignin573 with HY-340 niobic acid (eq 9), the linear and quadratic effects of the independent variables were significant. We can observe a positive linear effect and a negative quadratic effect on %HCA. Considering average operating conditions, the catalyst/biomass ratio (*x*_2_) significantly influenced %HCA and the deoxygenation of pyrolysis vapors. The effect of the catalyst/biomass ratio (*x*_2_) was approximately 14.77 times higher than the pyrolysis temperature (*x*_1_) on %HCA. Figure 6 presents the contour analysis applied to the results from the regression analysis of the experimental data using eqs 4–9.

**Figure 6:**
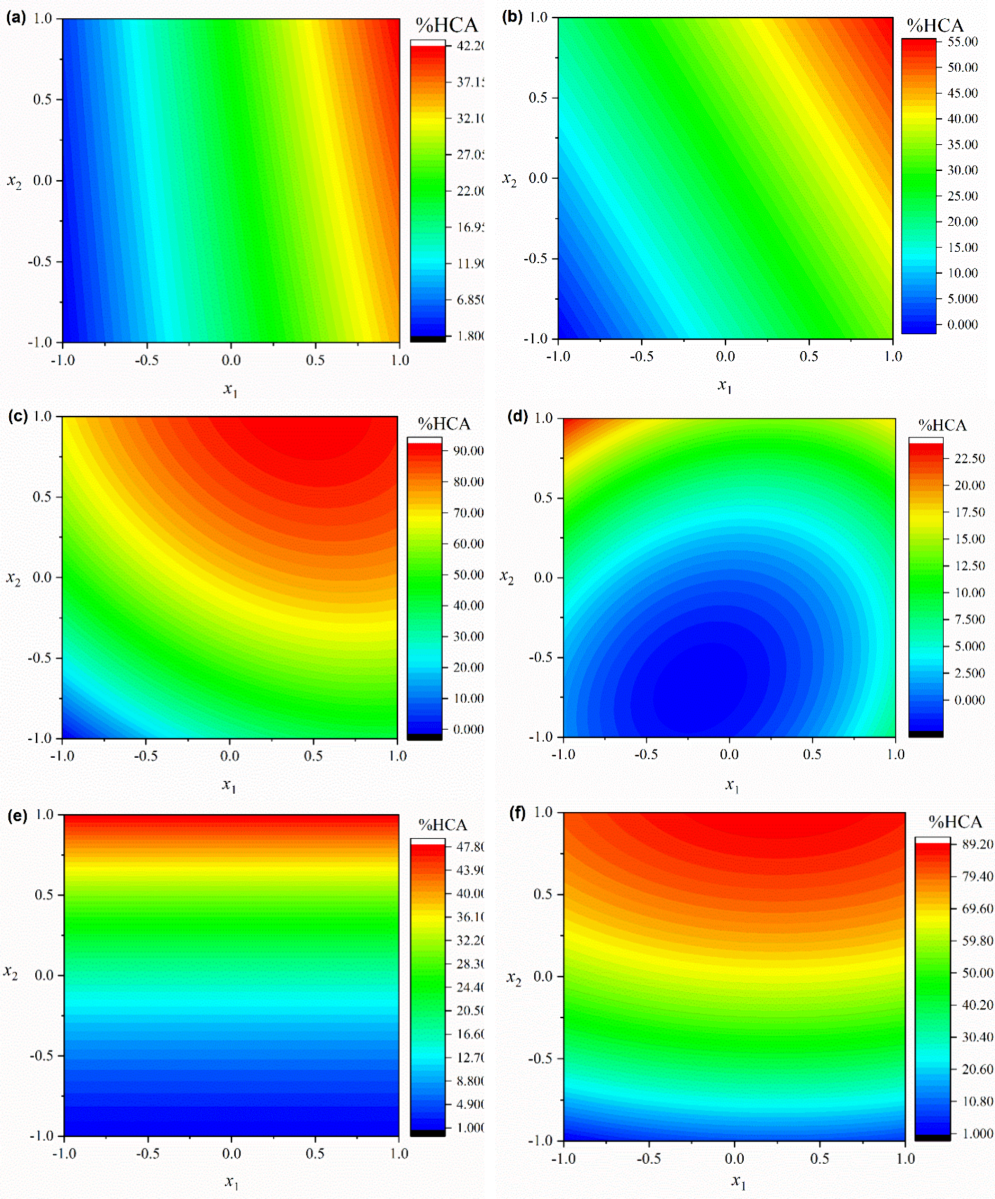
Contour analysis of the empirical equations for (a) Lignin493 (HZSM-5 zeolite), (b) Lignin533 (HZSM-5 zeolite), (c) Lignin573 (HZSM-5 zeolite), (d) Lignin493 (HY-340 niobic acid), (e) Lignin533 (HY-340 niobic acid), (f) Lignin573 (HY-340 niobic acid).

Figure 6 shows the influence of the independent variables on %HCA for each torrefied lignin. For Lignin493, Figure 6a indicates that the pyrolysis temperature (*x*_1_) strongly influenced %HCA. Figure 6d shows a positive effect of the catalyst/biomass ratio (*x*_2_) and the quadratic variables (*x*_1_^2^ and *x*_2_^2^) on %HCA. For Lignin533, Figure 6b indicates a positive effect of the independent variables on %HCA. The increase in independent variables increased %HCA, with the maximum %HCA achieved at the highest levels of these variables. Figure 6e shows that the pyrolysis temperature (*x*_1_) did not significantly influence %HCA.

For Lignin573, Figure 6c shows that both the pyrolysis temperature (*x*_1_) and catalyst/biomass ratio (*x*_2_) positively influenced %HCA and the deoxygenation of pyrolysis vapors. Thus, increasing independent variables can increase %HCA, achieving the maximum %HCA at the highest levels of these variables. Figure 6f indicates a positive effect of the independent variables on %HCA. However, the catalyst/biomass ratio (*x*_2_) has a higher influence on %HCA.

Based on the regression equations from the factorial experimental designs for torrefied Kraft lignin samples (eqs 4–9), we conducted reliability-based optimization using optimization differential evolution (MODE).^67^ The primary objective was to maximize the %HCA. Table 6 presents these optimization results using eqs 4–9.

**Table 6:** Optimization Results for the Catalytic Analytical Pyrolysis of Torrefied Lignin with HZSM-5 Zeolite and HY-340 Niobic Acid

material	eq	*x*_1_	*x*_2_	%HCA
Lignin493	4	1.00	1.00	42.01
Lignin533	5	1.00	1.00	55.46
Lignin573	6	0.45	1.00	92.35
Lignin493	7	–1.00	1.00	23.86
Lignin533	8		1.00	48.04
Lignin573	9	–0.25	1.00	89.40

Table 6 shows that Lignin 573 achieved the highest %HCAs. The catalyst/biomass ratio variable (*x*_2_) is obtained at the maximum level (+1) in all optimization results. These results are expected and align with the previous analyses for the catalytic analytical pyrolysis (Section 3.2). Since we aimed to reduce catalyst consumption in the pyrolysis experiments and reduce operating costs, we repeated the optimization to maximize %HCA while minimizing catalyst consumption. For this purpose, the optimization generates the population of solutions and, as a selection criterion, we considered those solutions in which %HCA was obtained by reducing selectivity by approximately 10% compared to the values in Table 6. Table 7 shows the optimization results for reducing 10% in the %HCA. We can observe that the catalyst/biomass ratio levels and the selectivity for aromatic hydrocarbons were reduced. For Lignin573, the reduction in the catalyst/biomass ratio maintained high selectivity for aromatic hydrocarbons and reduced operating costs.

**Table 7:** Optimization Results for the Catalytic Analytical Pyrolysis of Torrefied Lignin With HZSM-5 Zeolite and HY-340 Niobic Acid Reducing 10% in the Selectivity

material	equation	*x*_1_	*x*_2_	%HCA
Lignin493	4	1.00	–0.16	37.74
Lignin533	5	1.00	0.52	50.27
Lignin573	6	0.82	0.34	84.38
Lignin493	7	–0.99	0.90	21.35
Lignin533	8		0.91	44.53
Lignin573	9	0.30	0.52	81.76

Fast pyrolysis of industrial waste is still a technique that requires studies to reduce costs, mainly due to the cost of reaching high temperatures, such as 500 °C.^14^ Despite this, catalytic pyrolysis has proven to be an excellent technique for obtaining high-value-added products, contributing to the technique development and becoming a promising technique for waste treatment, as well as other techniques such as hydrogenolysis, solvent liquefaction, and fermentation.

### 3.4 Fast Pyrolysis in Bubbling Fluidized Bed Reactor

#### 3.4.1 Characterization of Bio-Oil Compounds

We characterized the bio-oil produced in the fast pyrolysis of Lignin573 at 823 K, analyzing the water content, pH, density, and viscosity. Table 8 presents the characteristics of the bio-oil from the fast pyrolysis of Lignin573 in a bubbling fluidized bed reactor.

**Table 8:** Characteristics of the Bio-Oil from the Fast Pyrolysis of Lignin573 in the Bubbling Fluidized Bed Reactor

property	bio-oil
pH	2.30 ± 0.01
density (g cm^–3^)	1.21 ± 0.00
water content (%)	24.31 ± 0.37
viscosity (313 K, cP)	17.43 ± 1.56

The bio-oil presented a low pH, probably due to oxygenated compounds that cause high acidity.^68^ This pH value aligns with those reported in the literature for wood oil (2.2–4.1) and crude bio-oil derived from wood (2.5). However, the acidic character of bio-oil may damage reactor equipment and facilities, hindering its application.^69,70^ In this study, the bio-oil density value agrees with wood oil (1.13–1.24 g cm^–3^) and crude bio-oil derived from wood (1.2 g cm^–3^),^67,68^ but it was higher than light fuel oil (0.85 g cm^–3^).^71^

The high water content is due to the original moisture in the raw material and the dehydration reactions during pyrolysis.^72^ Although the water present in bio-oil improves the fluidity characteristics, it reduces the calorific value, making ignition difficult and decreasing the combustion rate compared to diesel fuel.^68,73^ The water content of bio-oil from the fast pyrolysis of Lignin573 aligns with wood oil (13–33%) and crude bio-oil derived from wood (25%). Viscosity directly depends on the water content. It is important for pump and pipe sizing, and the operating temperature. The viscosity of pyrolysis oils is generally high due to the polymerization and condensation reactions that produce larger molecules and increase bio-oil viscosity.^74^ The bio-oil from fast pyrolysis had a calorific value of approximately 16–17 MJ kg^–1^ since it produces around 25%, by mass, of water that cannot be easily separated.^75^

Table 9 presents the ten main compounds of the bio-oil analyzed through GC/MS, according to the percentage of chromatographic peak area. Bio-oil is a complex mixture of oxygenated aliphatic and aromatic compounds from depolymerization and fragmentation reactions.^76^ We can observe the predominance of oxygenated compounds in bio-oil, particularly phenolic compounds. Oxygenated compounds are commonly detected in bio-oil from the pyrolysis of plant sources. They negatively affect bio-oil stability and energy content by reducing its calorific value and increasing its corrosivity.^75^ According to Imran et al.,^77^ the high acidity, high viscosity, corrosivity, repolymerization, and storage instability in bio-oil can be due to oxygenated compounds and water content. Therefore, the bio-oil quality must be improved to meet the strict specifications recommended for transportation of fuel.

**Table 9:** Main Compounds Present in the Bio-Oil From the Fast Pyrolysis of Lignin573 in a Bubbling Fluidized Bed Reactor

		% chromatographic peak area		
formula	RT^a^ (min)	average	standard deviation	compounds
C_7_H_8_O_2_	32.60	10.57	0.26	2-methoxyphenol (guaiacol)
C_8_H_10_O_2_	36.23	1.25	0.07	2-methoxy-3-methylphenol
C_8_H_10_O_2_	37.81	9.23	0.03	creosol
C_9_H_12_O_2_	41.85	6.56	0.11	4-ethyl-2-methoxyphenol
C_10_H_14_O_2_	44.63	1.15	0.10	1,4-dimethoxy-2,3-dimethylbenzene
C_10_H_14_O_2_	45.75	2.21	0.07	2-methoxy-4-propylphenol
C_8_H_10_O_3_	46.99	30.14	0.51	2,6-dimethoxyphenol (syringol)
C_9_H_12_O_3_	50.97	16.87	0.25	3,5-dimethoxy-4-hydroxytoluene
C_10_H_14_O_3_	54.05	11.87	0.17	1,2,3-trimethoxy-5-methylbenzene
C_10_H_12_O_4_	57.17	3.31	0.05	homosyringaldehyde

a Retention time.

The 2,6-dimethoxyphenol (syringol) was the main compound identified in the bio-oil, with a high percentage of chromatographic peak area. Santana Júnior et al.^21^ highlighted that syringol is the main compound responsible for the smoky aroma in the food industry. Based on the catalytic pyrolysis results and the analysis of bio-oil properties and compounds, it became clear that catalytic upgrading of bio-oil from fast pyrolysis is crucial to improve its suitability as a biofuel for transportation.^28^ Meng et al.^78^ studied the effect of torrefaction on the chemical composition of bio-oil from the fast pyrolysis of Pinus taeda in a fluidized bed at 773 K. The GC/MS analysis showed a high content of lignin compounds, indicating good potential to produce phenolic-based chemicals. The authors concluded that torrefaction is an effective pretreatment strategy for fast pyrolysis to produce high-quality bio-oil.

#### 3.4.2 Comparison between the Composition for the Compounds from Analytical Pyrolysis and Bio-Oil

We compared the compounds generated in the analytical pyrolysis of raw Kraft lignin and torrefied Kraft lignin (Lignin573) at 823 K with the compounds in the bio-oil produced in the fast pyrolysis of Lignin573 (823 K) in a bubbling fluidized bed reactor. Figure 7 shows the compounds of these three scenarios. In the three experimental conditions, oxygenated compounds were predominant due to phenolic compounds. The analytical pyrolysis of raw lignin produced 52.66% oxygenated compounds; the analytical pyrolysis of Lignin573 yielded 64.87%, and the bio-oil resulted in 78.15%. In the literature, the differences in the composition of the compounds of the analytical pyrolysis of the raw and torrefied Kraft lignin compared with the composition of the bio-oil produced in the bubbling fluidized bed reactor can be attributed to the secondary reactions occurring during the fast pyrolysis in the fluidized bed reactor since the residence time of the pyrolysis vapors in the analytical pyrolysis is shorter than that in the fast pyrolysis unit. Thus, the secondary reactions are neglected in analytical pyrolysis.^28,30^

**Figure 7:**
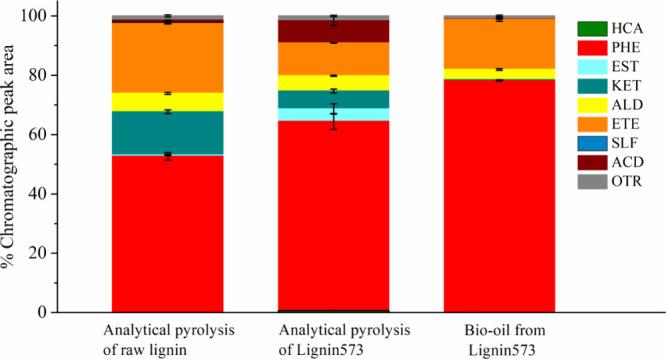
Comparison among the compounds in the products from analytical pyrolysis of raw and torrefied Kraft lignin with the compounds in bio-oil. Hydrocarbons (HCA), phenols (PHE), esters (EST), ketones (KET), aldehydes (ALD), ethers (ETE), sulfonated (SLF), acids (ACD), and others (OTR)

These differences in composition can also be attributed to the polarity effects of the compounds in the mobile phase (methanol) and the stationary phase of the chromatographic column. Methanol is a polar solvent used in the extraction and dilution of bio-oil for analysis in the chromatograph. Figure 7 shows that the bio-oil from Lignin573 did not present a high acid formation. However, Table 8 shows that the bio-oil presented high acidity, which can be attributed to oxygenated compounds, especially phenolic compounds. For De Castro et al.,^79^ the bio-oil acidity is due to oxygenated compounds, such as carboxylic acids, phenols, cresols, ketones, and aldehydes.

## 4 Conclusions

We performed analytical pyrolysis using the Py-GC/MS system at 723, 823, and 923 K for the pyrolysis of raw and torrefied Kraft lignin samples. The pyrolysis vapors produced in analytical pyrolysis showed high levels of oxygenated compounds, mainly phenolics. For the raw Kraft lignin, the maximum production of phenolic compounds was 74% at 923 K. There was no production of aromatic hydrocarbons for the raw and torrefied Kraft lignin samples in the analytical pyrolysis, except for Lignin573, which showed a small production at the three temperatures studied. HZSM-5 zeolite considerably increased the production of aromatic hydrocarbons, obtaining a maximum of 57.84% with a catalyst/biomass ratio of 10:1 at 923 K. For HY-340 niobic acid, the maximum production of aromatic hydrocarbons was 87.24 and 86.75% at 823 and 923 K, respectively, with a catalyst/biomass ratio of 10:1.

We also performed a factorial experimental design with HZSM-5 zeolite and HY-340 niobic acid to evaluate the effects of the pyrolysis temperature and catalyst/biomass ratio on the %HCA. These results showed that the maximum %HCA values were 92.84 and 90.02% for Lignin573 with HZSM-5 zeolite and HY-340 niobic acid, respectively, with a catalyst/biomass ratio of 5:1. Optimizations are performed to maximize the production of aromatic hydrocarbons, and the optimum points were at the maximum level of the catalyst/biomass ratio, as expected. The catalytic analytical pyrolysis of lignin shows that the use of catalysts plays an important role in the deoxygenation of pyrolysis vapors from lignin. Therefore, the maximum production of aromatic hydrocarbons can be obtained by torrefaction associated with catalysts in pyrolysis. In future work, a techno-economic or catalyst recycling assessment could be performed to evaluate the applicability of high catalyst-to-biomass ratios at industrial scales. In addition, more in-depth chemical analyses can be performed to evaluate the liquid, solid, and gaseous products of the fast pyrolysis of kraft lignin in bubbling fluidized bed reactors.

## Supplementary Material



## Notes

Multiple regression tables with parameter values and significance levels (p-level) for the full and reduced model for Lignin493 with HZSM-5, Lignin493 with HY-340, Lignin533 with HZSM-5, Lignin533 with HY-340, Lignin573 with HZSM-5, and Lignin573 with HY-340 (PDF)
