# Supplementary material for: Pyrolysis of Kraft Lignin: The Effect of HZSM‑5 and HY-340 Catalysts and Torrefaction Pretreatment
Source: ACS Omega. 2025 Jun 30;10(27):29705–20. doi: 10.1021/acsomega.5c03569 (PMC12268729; doi:10.1021/acsomega.5c03569)
Supplement: Supplementary file 1 [file ao5c03569_si_001.pdf]

Supporting Information

Pyrolysis of Kraft lignin: the effect of HZSM-5 and HY-340 catalysts and torrefaction pretreatment

Anderson L. de Menezes[a]; *Alvaro E. C. Souza*[b]; *Daniel A. Cerqueira*[c*]; *Cássia R. Cardoso*[d*]; *Luiz G. M. Vieira*[a]

[a]*Faculty of Chemical Engineering. Federal University of Uberlândia. 38408-100. Uberlândia. MG. Brazil*

[b]*Multicenter Chemistry Graduate Program of Minas Gerais State. Federal University of Triângulo Mineiro. 38064-200. Uberaba. MG. Brazil*

[c]*Department of Chemistry. Federal University of Triângulo Mineiro. 38064-200. Uberaba. MG. Brazil*

[d*]*Department of Food Engineering. Federal University of Triângulo Mineiro. 38064-200. Uberaba. MG. Brazil*

[*]Corresponding author: Cássia R. Cardoso. Email: cassia.cardoso@uftm.edu.br

Table S1: Regression parameters for Lignin493 with HZSM-5

Coefficient	Full model		Reduced model	
	Value	*p*-level	Value	*p*-level
β_0	21.37778	0.000000	20.77722	0.000000
β_1	17.59167	0.000000	17.59167	0.000000
β_2	2.47750	0.001045	2.47750	0.000907
β_{11}	0.61833	0.547764	–	–
β_{22}	-1.51917	0.154477	–	–
β_{12}	1.66000	0.036814	1.66000	0.037886

Table S2: Regression parameters for Lignin493 with HY-340.

Coefficient	Full model		Reduced model	
	Value	*p*-level	Value	*p*-level
β_0	0.72111	0.743181	–	–
β_1	-1.47750	0.233607	–	–
β_2	8.12167	0.000017	8.12167	0.000014
β_{11}	6.26583	0.009696	6.26583	0.010130
β_{22}	6.27833	0.009586	6.27833	0.010013
β_{12}	-3.19000	0.047179	-3.19000	0.049670

Table S3: Regression parameters for Lignin533 with HZSM-5

Coefficient	Full model		Reduced model	
	Value	*p*-level	Value	*p*-level
β_0	24.41000	0.000000	26.92667	0.000000
β_1	17.94583	0.000000	17.94583	0.000000
β_2	10.58417	0.000001	10.58417	0.000000
β_{11}	3.26750	0.133753	–	–
β_{22}	0.50750	0.806973	–	–
β_{12}	-0.70000	0.634857	–	–

Table S4: Regression parameters for Lignin533 with HY-340.

Coefficient	Full model		Reduced model	
	Value	*p*-level	Value	*p*-level
β_0	13.92722	0.002580	16.56833	0.000034
β_1	-2.53833	0.231319	–	–
β_2	23.64500	0.000000	23.64500	0.000000
β_{11}	3.96167	0.278072	–	–
β_{22}	7.82667	0.044428	7.82667	0.040187
β_{12}	0.77625	0.758297	–	–

Table S5: Regression parameters for Lignin573 with HZSM-5

Coefficient	Full model		Reduced model	
	Value	*p*-level	Value	*p*-level
β_0	72.1661	0.000000	72.1661	0.000000
β_1	15.3875	0.000000	15.3875	0.000000
β_2	29.7717	0.000000	29.7717	0.000000
β_{11}	-11.3242	0.000566	-11.3242	0.000566
β_{22}	-11.8667	0.000387	-11.8667	0.000387
β_{12}	-5.2375	0.010319	-5.2375	0.010319

Table S6: Regression parameters for Lignin573 with HY-340.

Coefficient	Full model		Reduced model	
	Value	*p*-level	Value	*p*-level
β_0	65.1289	0.000000	65.1289	0.000000
β_1	2.7325	0.014477	2.7325	0.021731
β_2	40.3308	0.000000	40.3308	0.000000
β_{11}	-5.5408	0.005857	-5.5408	0.009271
β_{22}	-16.2608	0.000000	-16.2608	0.000001
β_{12}	2.2238	0.082099	–	–
